# Infection-stage adjusted dose of beta-lactams for parsimonious and efficient antibiotic treatments: A *Pasteurella multocida* experimental pneumonia in mice

**DOI:** 10.1371/journal.pone.0182863

**Published:** 2017-08-04

**Authors:** Maleck V. Vasseur, Marlene Z. Lacroix, Pierre-Louis Toutain, Alain Bousquet-Melou, Aude A. Ferran

**Affiliations:** Toxalim (Research Centre in Food Toxicology), Université de Toulouse, INRA, ENVT, INP-Purpan, UPS, Toulouse, France; University of Illinois at Urbana-Champaign, UNITED STATES

## Abstract

In this study, the impact of infection stage on clinically and microbiologically efficacious doses and on antibiotic consumption was assessed during a naturally evolving infectious disease, using an original mouse model of pulmonary infection produced by air-borne contamination. When *Pasteurella multocida* was administered as pathogenic agent to immunocompetent mice, 60% of the animals exhibited clinical symptoms of pneumonia 2 to 4 days after bacterial contamination of the lungs. Two beta-lactam antibiotics were evaluated: amoxicillin and cefquinome, a fourth generation cephalosporin developed for food animals. First, a pharmacokinetic study was performed in infected mice to determine the exposure to amoxicillin or cefquinome required to treat clinically affected animals, based on the targeted values of PK/PD indices for beta-lactams. We then confirmed that these doses resulted in a 100% clinical cure rate in animals exhibiting clinical signs of infection and harboring a high pathogenic inoculum. More interestingly, we also showed that the same 100% clinical cure could be obtained in our model with 10-fold lower doses in animals at pre-patent stages of infection i.e. when harboring a low pathogenic inoculum. At the group level, antimicrobial drug consumption was reduced by treating animals at an early stage of the infection course with a pre-patent tailored dose. These results suggest that early treatment with a dose suitably adjusted to the stage of infection might help to reduce both overall antibiotic consumption and resistance selection pressure in the animals and in the environment.

## Introduction

Bacterial resistance to antimicrobials has become a worldwide problem and an increasing threat to human health. The main cause is considered to be the inadequate use of antimicrobial agents leading to exposure of bacteria to antibiotics favoring selection of bacterial resistance, not only at the site of infection but also in commensal bacteria, including those harbored by the intestinal microbiota [1]. In this context, various antimicrobial strategies are being proposed, the aim being to design dosage regimens that not only attain clinical efficacy, but also minimize the emergence and spread of resistance [2,3]. One promising strategy to improve antimicrobial use could be to optimize drug dosing regimens as a function of the pathogenic inoculum size. *In vitro* studies and to some extent *in vivo* models of infection have indeed shown that antimicrobial treatments targeting low pathogen inoculums are efficacious at doses much lower than those required to eradicate high inoculums [4–12]. Moreover, we have already shown that the low doses of cefquinome, a fourth generation cephalosporin, sufficient to eradicate a low inoculum of *Klebsiella pneumoniae* in a rat model of infection led to lower selection of intestinal *E*. *coli* carrying Extended Spectrum Beta-Lactamase (ESBL) [12]. These results support the hypothesis that, in the case of a low bacterial pathogen load, reducing the dose could simultaneously ensure a cure of the infection and avoid the selection and amplification of ESBL-carrying Enterobacteria in the gut microbiota. Thus the strategy consisting of proposing an infection-stage adjusted dose (or inoculum-size adjusted dose), could potentially help to preserve public health by reducing the antimicrobial selection pressure on commensal bacteria. In veterinary medicine, the optimization of drug dosage has to consider the group level, the overall aim being to reduce antimicrobial consumption in food-producing animals. Bovine Respiratory Disease (BRD), a major disease in young beef cattle, has a huge economic impact on the farming industry due to production losses and the costs of treatment and prevention [13]. Two therapeutic strategies are commonly implemented by veterinarians and farmers to deal with this typically collective disease which affects, on average, 15% of the animals in a herd but up to 80% in some herds [14,15]. The first therapeutic approach, similar to the one adopted in human medicine, consists of treating only the affected animals, regardless of the severity of their clinical signs [15,16]. Although this “late” approach limits antibiotic consumption and exposure, it can also lead to extensive pulmonary damage in affected animals and to spread of the disease within the herd [16,17]. Metaphylaxis, the second approach, is therefore more frequently adopted as an early BRD treatment and consists of treating the entire cohort of animals when only a few express clinical signs of BRD [18,19]. This approach has the advantages of ensuring both a good survival rate of the cohort and ease of use [18] but, compared to the first individual approach, has the disadvantage of exposing animals to antibiotic consumption even when this is not required. For regulatory reasons, both approaches currently involve administration of the same individual labelled doses with no consideration of the stage of the infection. This means that, with the metaphylaxis approach, drug consumption is probably higher than required to actually eradicate the pathogens.

The objective of the present study was to see if a reduction of the dose administered to pre-patent animals could ensure the efficacy of antimicrobial treatments and also reduce overall antibiotic consumption at the individual and group levels. The first aim was to use a mouse model of air-borne lung infection to explore the impact of the time of therapeutic intervention during the natural time course of an infectious disease caused by *Pasteurella multocida*, one of the pathogenic bacteria responsible for BRD in cattle. The second aim was to perform a PK study of amoxicillin and cefquinome in infected mice to select the dosage regimens for subsequent testing and finally, the main objective was to compare the impact of amoxicillin or cefquinome doses, adjusted to the bacterial load, on collective and early treatments *versus* targeted and late treatments by measuring the clinical and microbiological cures and antimicrobial consumption.

## Materials and methods

### Bacterial strain

A strain of *Pasteurella multocida*, isolated from the trachea of a pig with clinical symptoms of a bacterial lung infection, was used as the test bacteria.

### Antimicrobial drugs

Powder of amoxicillin sodium (Clamoxyl^®^ 1g for injectable suspension, GSK, France) and powder of cefquinome sulphate (Cobactan^®^ IV-IM 4.5%, Intervet, France) were used for MIC determinations. Amoxicillin trihydrate Long Acting (LA) (Clamoxy^®^ LA, 150mg/mL, Pzifer Santé Animale, France) and cefquinome sulphate LA (Cobactan^®^ LA 7.5%, 75mg/mL, Intervet, France) were used for *in vivo* experiments. Before administration to animals, the long-acting solutions were first diluted at concentrations from 1 to 10 mg/mL for amoxicillin and from 0.2 to 2 mg/mL for cefquinome in corn oil (Sigma, France) to obtain a sufficient volume for injection (≈100μL/mouse).

### MIC determination

MIC of amoxicillin and cefquinome on the *P*. *multocida* strain used in the *in vivo* experiments were determined in triplicate by broth microdilution according to the CLSI reference methods [20]. Briefly, a bacterial suspension, diluted in MHB to give a final organism density of 5.10^5^ CFU/mL, was added to wells of a microtiter plate containing serial 2-fold dilutions of amoxicillin or cefquinome. Growth was recorded after 18 h of incubation at 35°C.

### Animals

Female Swiss mice (Charles River Laboratories, L’Arbresle, France) were used for all studies. The experiments were conducted in accordance with the Guide for the Care and Use of Laboratory Animals as adopted and promulgated by the U.S. National Institutes of Health and under the agreement number TOXCOM/0022/AF for animal experimentation from the French Ministry of Agriculture (Ethic Committee C2EA-86). The clinical status of the mice was checked twice a day on the seven days after infection. Mice were considered as sick when they expressed at least one symptom among closed set eyes, low mobility, quilted coat, slight hunching and moderate dyspnea. If, at any time during the experiment, the mice were unable to move, they were immediately and humanely sacrificed by intraperitoneal injection of sodium pentobarbital (Dolethal^®^, Vetoquinol, France).

### Lung aerial infection model

Female Swiss mice were placed in an Inhalation Exposure System (IES, model 099CA4224, Glas-Col^®^, IN, USA) and 10 mL of a *P*. *multocida* suspension containing 5.10^9^ CFU/mL were loaded in the Nebulizer-Venture Unit. The IES was programmed as follows: Preheat: 15 min, Nebulizing: 120 min, Cloud Decay: 30 minand Decontamination: 15 min.

### Pharmacokinetics

Thirty female Swiss mice were used for the pharmacokinetic study of amoxicillin and cefquinome. Mice were infected with *P*. *multocida* as previously described and 24 hours later, a single dose of amoxicillin or cefquinome was subcutaneously injected between the shoulders of each animal. For amoxicillin, a diluted solution of 10 mg/mL was prepared and each mouse received a 50 mg/kg dose. For cefquinome, a diluted solution of 1 mg/mL was prepared and each mouse received a 5mg/kg dose.

Groups of 3 mice were anesthetized with 3% isoflurane (Aerrane^®^, Baxter France) in O_2_ at 5 min and 0.5, 1, 2, 3, 4, 6, 8, 12 or 24 h after dosing. Terminal blood samples (one sample *per* animal) were collected by intracardiac puncture and centrifuged at 7000g for 10 min at 4°C. Plasma samples were stored at -80°C until assay.

#### Amoxicillin assays

Plasma amoxicillin concentrations were assayed by LC/MS/MS using a Thermofinnigan Surveyor^®^ HPLC system with a LCQ Deca XP Max^®^ ion trap mass spectrometer (Thermo Electron Corporation, Waltham, Mass, USA). The limit of quantification (LOQ) was 20ng/mL and accuracy varied from 98 to 106%. The intra-day and inter-day coefficients of variation for precision ranged from 5.22 to 7.04% and from 6.86 to 11.8% respectively.

#### Cefquinome assays

Plasma cefquinome concentrations were assayed with an Acquity ultra performance liquid chromatography system (UPLC^®^) coupled to a Xevo^®^ triple quadrupole mass spectrometer (Waters, Milford, MA, USA). The LOQ was 10ng/mL and accuracy varied from 96 to 104%. The intra-day and inter-day coefficients of variation for precision ranged from 6.72 to 11.7% and from 10.7 to 15.1% respectively.

#### Data analysis

A naïve pooled-data approach was used to fit the antibiotic concentrations *versus* time profiles to a bicompartmental model with extravascular administration. Fitting was done with WinNonlin^®^ version 5.3 (Pharsight Corporation^®^, Mountain View, CA, USA). The inverse of the square of the predicted values was used as weighting scheme.

### Kinetics of *P*. *multocida* growth in mouse lungs

Forty female Swiss mice were infected by *P*. *multocida* with the IES as previously described. Mice were sacrificed humanely by intraperitoneal injection of sodium pentobarbital (Dolethal^®^, Vetoquinol, France) at 0 (3 mice), 24 h (3), 48 h (2), 72 h (5), 96 h (8), 120 h (6) and 144 h (7 mice) to count the bacteria in the lungs. In addition, lung bacteria were also counted in the mice sacrificed because of severe clinical signs (immobility). The number of mice at each time depended on the disparity of clinical signs in the group since our aim was to sacrifice at least 2 to 3 mice with similar clinical status at each time. Lung bacterial load was determined as follows: the lungs of each mouse were aseptically removed and homogenized in 10 mL of 0.9% NaCl. The homogenates were centrifuged at 3000g for 10 min then the pellets were resuspended in 2.5 mL of 0.9% NaCl. Ten microliters of successive 10-fold dilutions of homogenates were then plated in triplicate on MH-agar. The colonies were counted after incubation for 24 h at 37°C. If the colonies were too small, incubation was continued for a further 24 h. The lowest level of detection was 100 CFU/lung and below this level bacteria were considered eradicated.

### Antibiotic treatments

**Fig 1:**
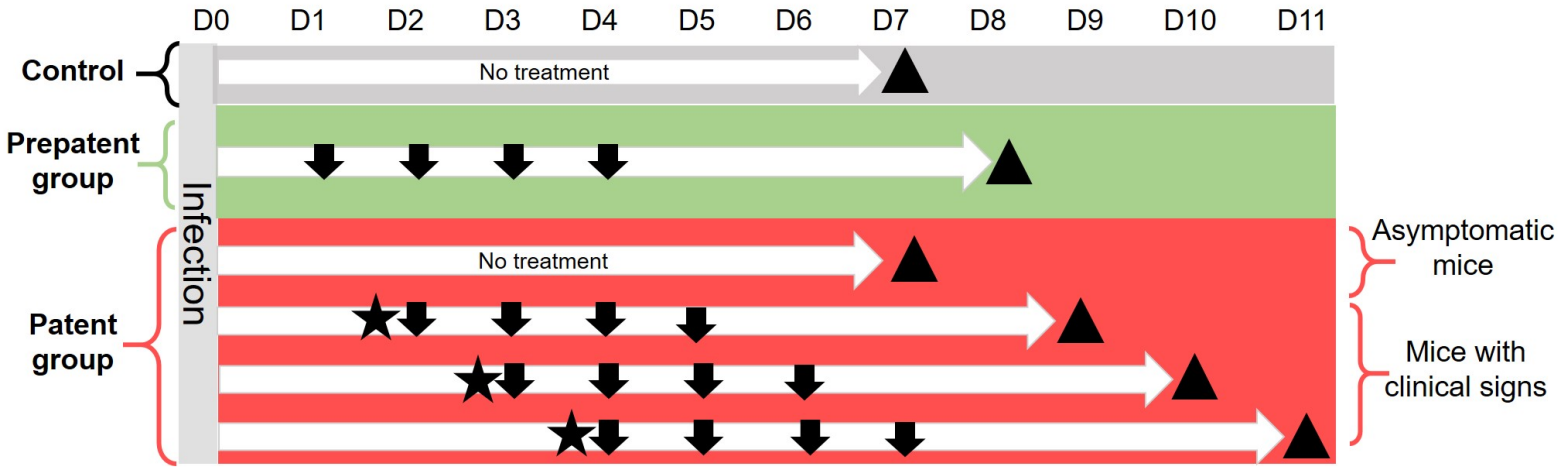
Time-course of the administered treatments. Mice were infected at Day 0 and were treated or not with amoxicillin or cefquinome depending on the treatment phase (control, pre-patent or patent treatment) and on the group. Mice were sacrificed seven days after the challenge or the beginning of treatment (triangle). Arrows represent the administrations of amoxicillin or cefquinome (each treatment corresponded to 4 daily administrations). The administered doses depended on the treatment phase (control, pre-patent or patent treatment) and on the group. The star indicates that at least one clinical symptom was observed and that the mouse was considered as sick.

The time-course of the administered treatments, depending on the groups of mice, is represented in Fig 1.

#### Control group

Twenty mice were infected with *P*. *multocida*, using the IES. The clinical status of each animal was then evaluated and recorded twice daily for seven days in the absence of any antimicrobial treatment.

#### Pre-patent phase treatments

Four groups of ten mice were infected with *P*. *multocida*, using the IES. Twenty-four hours later, all mice in each group received an antibiotic treatment as a single daily subcutaneous injection between the shoulders for four days: two groups received cefquinome (0.5 or 1 mg/kg/day) and two groups received amoxicillin (2.5 or 5 mg/kg/day). The clinical status of each animal was evaluated and recorded twice daily for seven days after the bacterial challenge.

#### Patent phase treatments

Six groups of ten mice were infected with *P*. *multocida*, using the IES. The mice were observed and clinically assessed twice daily and the antibiotic treatment (consisting of a daily subcutaneous injection between the shoulders for four days) was launched as soon as an animal expressed clinical signs of infection (closed set eyes, low mobility, quilted coat, slight hunching, or moderate dyspnea). Three groups received cefquinome (1, 5 or 10 mg/kg/day) and three groups received amoxicillin (5, 25 or 50 mg/kg/day). Mice which had not expressed any clinical symptom during the seven days after the challenge were not treated. The clinical status of all animals (treated and untreated) was evaluated and recorded twice daily for seven days after the challenge.

In all experiments (control or treatments), mice were sacrificed seven days after the challenge or the beginning of treatment by administering an intraperitoneal injection of sodium pentobarbital (Dolethal^®^, Vetoquinol, France) and the lung bacterial load was determined with the same procedure as previously described.

## Results

### Susceptibility testing

The MIC of amoxicillin and cefquinome for the tested *P*. *multocida* strain were 0.125 and 0.016 μg/mL respectively.

### Pharmacokinetics of amoxicillin and cefquinome in mice and selection of dosage regimens

**Fig 2:**
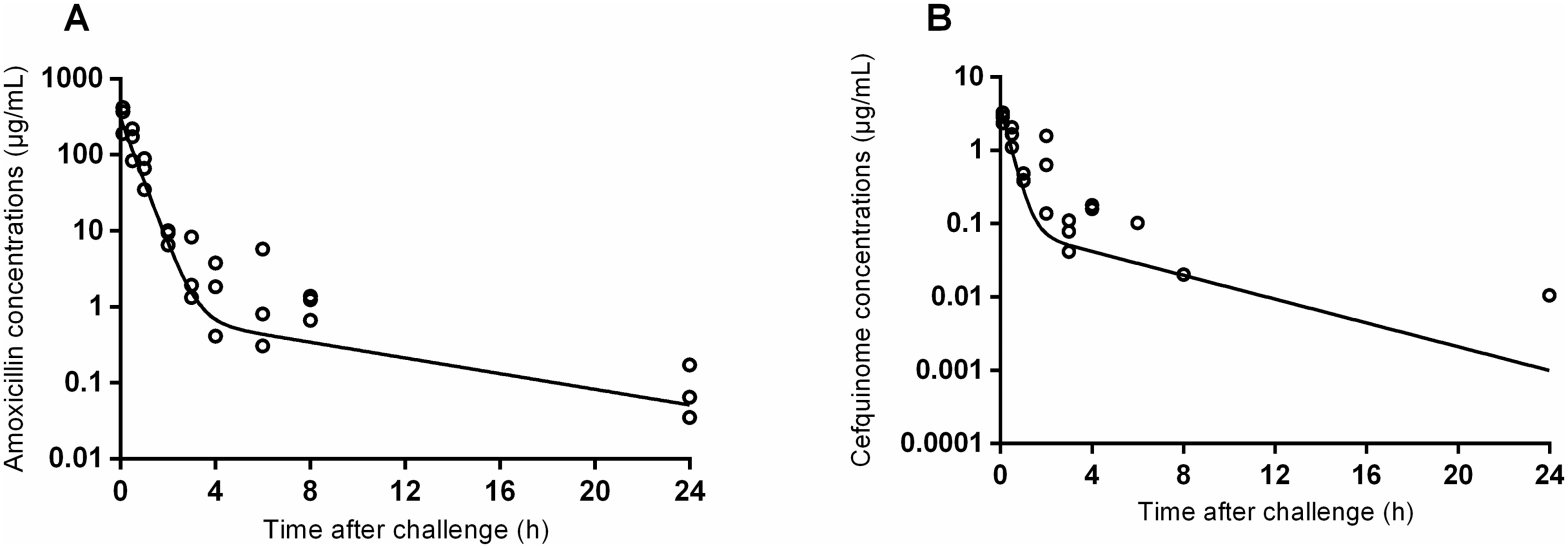
Amoxicillin and cefquinome pharmacokinetics in mice. Observed (○) and predicted (-) plasma antibiotic concentrations *versus* time in mice (n = 3 at each time point) after a single subcutaneous administration. A) Amoxicillin, 50 mg/kg. B) Cefquinome, 5 mg/kg. Cefquinome concentrations were below the LOQ for one mouse at 4 h, 2 mice at 6, 8 and 24 h and 3 mice at 12 h.

The observed and predicted plasma concentrations following a single subcutaneous injection of 50 mg/kg amoxicillin and of 5 mg/kg cefquinome (long-acting formulations) in infected but asymptomatic mice are shown in Fig 2 (raw data in S1 Table). The estimated pharmacokinetic parameters are presented in Table 1. The areas under the plasma concentration *versus* time curves (AUC _[0-∞]_) for amoxicillin and cefquinome were 163 and 2 μg.h/mL, respectively. The corresponding elimination half-lives (t_1/2_) were 5.8 and 3.4h. The average plasma concentrations over 24h were 6.79 and 0.083 μg/mL for amoxicillin and cefquinome, respectively. The times during which the plasma antibiotic concentrations were above the corresponding MIC (T_>MIC_) were 16.5 and 12.9 h for amoxicillin and cefquinome, respectively (Table 2).

**Table 1:** Pharmacokinetic and pharmacodynamic parameters of amoxicillin and cefquinome in mice after subcutaneous injection.

Administered antibiotic	Amoxicillin	Cefquinome
**Dose (mg/kg)**	**50**	**5**
AUC_[0-∞]_ (μgxh/mL)	163	2
C_max_ (μg/mL)	277	2.8
T_1/2elim_ (h)	5.8	3.4
CL/F (mL/h/kg)	306	2430
C _average_ (μg/mL)	6.79	0.083
MIC for *P*.*multocida* (μg/mL)	0.125	0.016

AUC (Area Under the plasma concentration Curve), C_max_ is the observed maximum plasma concentration, T_1/2elim_ is the terminal elimination half life, CL/F is the apparent plasma clearance (Dose/ AUC_[0-∞]_) and C _average_ is the mean antibiotic concentration over 24 hours.

**Table 2:** Values of the PK/PD index T_>MIC_ for different dosing regimens of amoxicillin and cefquinome.

Administered antibiotic	Amoxicillin			Cefquinome		
**Dose (mg/kg)**	**50**	**25**	**5**	**10**	**5**	**1**
T_>MIC_ (h)	16.5	10.7	3.2	12.9	9.2	1.9
T_>MIC_ (% of dosing interval)	69	45	13	54	38	8

Assuming dose-proportionality for both drugs, different plasma amoxicillin and cefquinome concentration profiles were simulated to determine the doses for which T_>MIC_ would attain the target value of 50% of the dosing interval (T_>MIC_ = 50%) [21]. Based on this targeted value of the PK/PD index, the dosing regimens expected to attain the PK/PD target were 50 mg/kg/day for amoxicillin and 10 mg/kg/day for cefquinome.

### Growth of *P*. *multocida inocula* in mice

**Fig 3:**
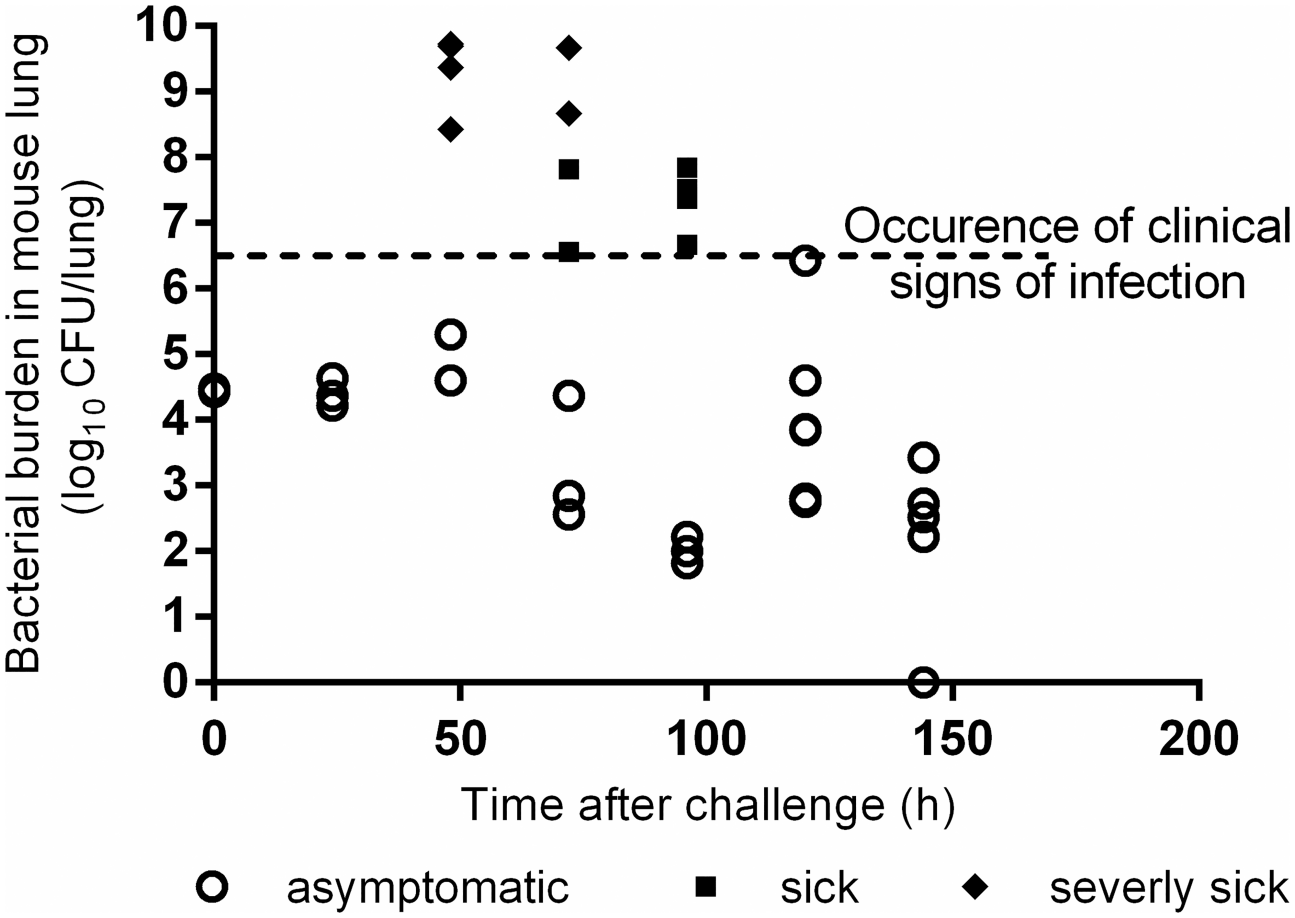
Growth of *Pasteurella multocida* inocula *versus* time (h) in mouse lungs after an aerial challenge. Symbols represent pathophysiological status of mice: asymptomatic mouse (empty circle), sick mouse (full square), severely sick mouse (full diamond).

The time-course of bacterial growth in the lungs of control mice is shown in Fig 3 (raw data in S2 Table). The bacterial load actually present in the lungs at the end of the cycle of infection in the IES^®^ (t = 0h) was highly reproducible at around 4.5 log_10_ CFU/lung (3 mice). Twenty-four hours after the inhalation challenge, all mice were asymptomatic and the total bacterial population remained steady, in the range of 4.2–4.6 log_10_ CFU/lung (3 mice). Thus, the treatments performed at 24h post-challenge in the subsequent pre-patent phase group were considered to have been administered during the pre-patent phase of the infection (regardless of its future evolution).

From 48 hours after the challenge, groups of mice could be distinguished with no clinical signs, mice with slight clinical signs of infection (closed set eyes, low mobility, quilted coat or hunched) and very few mice with severe clinical signs (immobility, severe dyspnea) that were sacrificed. From 0 to 7 days, the bacterial load in the lungs of mice with no clinical sign of infection at the time of sacrifice was below 5 log_10_ CFU except in 2 /25 mice. The bacterial loads in the lungs of all mice with mild clinical signs ranged from 6 to 8 log_10_ CFU (8 mice) and were higher than 8 log_10_ CFU in the 8 mice with severe clinical signs.

### Clinical and microbiological outcomes

#### Control group

For the control group with no antimicrobial treatment, 60% (12/20) of the mice presented clinical signs of infection which all became severe and led to sacrifice within seven days after the challenge. Twenty-five percent (5/20) of the challenged mice did not present any clinical signs of infection but a mean bacterial load of 4.73±1.12 log_10_ CFU could be detected in their lungs at day 7 after the challenge. Fifteen percent (3/20) of the challenged mice did not have any clinical symptoms or detectable bacteria in the lungs at seven days post-challenge.

#### Pre-patent phase treatments

In the four pre-patent phase groups, no clinical sign was observed in any animal at the beginning of the treatments (24h post-challenge).

**Fig 4:**
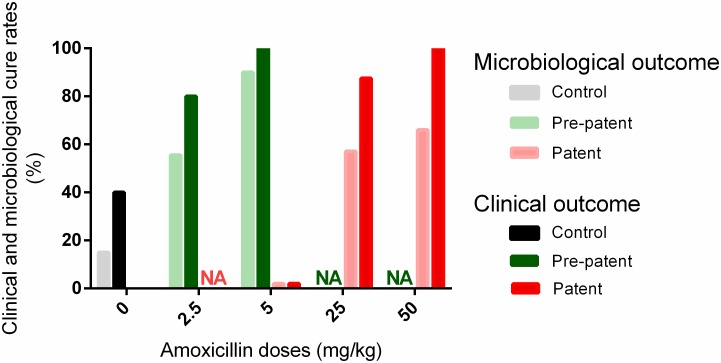
Clinical and microbiological cure rates with amoxicillin. Clinical (dark bars) and microbiological (light bars) cure rates of mice after no treatment (grey/black), early (green) or late (red) treatments with different doses of amoxicillin (2.5, 5, 25, or 50 mg/kg). Rates were calculated for all the mice of the group in control and early treatments (as all the mice in a given group were subjected to the same protocol) whereas cure rates for the late treatments were only calculated from the 60 to 70% of mice treated with amoxicillin NA: not assessed.

In the two amoxicillin groups (doses of 2.5 or 5mg/kg/day), 80% and 100% of the treated mice, respectively, did not present any clinical sign of infection during the six days after beginning the treatment (7 days post-challenge) (Fig 4, S3 Table). In addition, a microbiological cure (no bacteria detected in lungs) was obtained in 55.5% and 90% of the treated mice at day 7 after the bacterial challenge for the 2.5 and 5 mg/kg/day doses, respectively (Fig 4, S3 Table). The mean bacterial load in mice presenting no microbiological cure was 3.78±2.20 log_10_ CFU/lung (4 mice) and 2.56 log_10_ CFU/lung (1 mouse) for the 2.5 and 5mg/kg/day doses, respectively.

**Fig 5:**
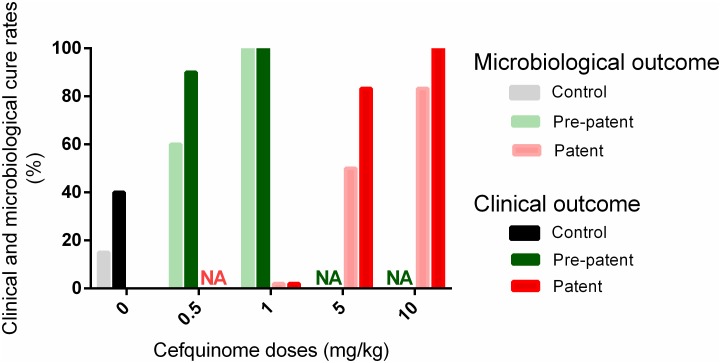
Clinical and microbiological cure rates with cefquinome. Clinical (dark bars) and microbiological (light bars) cure rates of mice after no treatment (grey/black), early (green) or late (red) treatments with different doses of cefquinome (0.5, 1, 5, or 10 mg/kg). Rates were calculated for all the mice of the group in control and early treatments (as all the mice in a given group were subjected to the same protocol) whereas cure rates for the late treatments were only calculated from the 60 to 70% of mice treated with cefquinome NA: not assessed.

In the two cefquinome groups (0.5 or 1mg/kg/day doses), 90% and 100% of the treated mice, respectively, did not present any clinical sign of infection for six days after the beginning of treatment (7 days post-challenge)(Fig 5, S3 Table). In addition, a microbiological cure was obtained 7 days after the bacterial challenge in 60% and 100% of the mice treated with 0.5 and 1mg/kg/day doses, respectively (Fig 5, S3 Table). The mean bacterial load in mice presenting no microbiological cure was 3.92±2.51 log_10_ CFU/lung (4 mice) for the 0.5mg/kg/day dose.

#### Patent phase treatments

Sixty to seventy percent of the infected mice in the six groups that were destined to receive the patent phase treatments, presented clinical signs of infection after the challenge, and were then treated with amoxicillin or cefquinome. The 30–40% of animals in these groups which did not present clinical signs were not treated with antimicrobial drugs.

All clinically affected mice that received the 5 mg/kg/day dose of amoxicillin were sacrificed due to severe clinical signs before day 7 after the challenge.

Among the mice treated with 50 or 25 mg/kg/day amoxicillin, 100% and 85.7% were clinically cured and 66.6% and 57.1% were microbiologically cured (Fig 4, S3 Table) at day 7 after the challenge. The bacterial loads in mice which were not microbiologically cured were 3.71±0.41 log_10_ CFU/lung (2 mice) and 4.11±2.73 log_10_ CFU/lung (4 mice) for the 50 and 25 mg/kg/day doses, respectively.

All clinically affected mice that received the 1 mg/kg/day dose of cefquinome were sacrificed due to severe clinical signs before day 7 after the challenge.

Among the mice treated with the 10 and 5 mg/kg/day doses of cefquinome, 100% and 83.3% were clinically cured and 83.3% and 50% were microbiologically cured, respectively (Fig 5, S3 Table) at day 7 after the challenge. The bacterial loads in mice that received the 10 and 5 mg/kg/day doses, and which presented no microbiological cure, were 3.42 log_10_ CFU/lung (1 mouse) and 4.46±1.75 log_10_ CFU/lung (4 mice) respectively.

## Discussion

In this study, we observed that antimicrobial doses could be adjusted to the stage of infection without compromising the clinical outcome and that administering a suitably-adjusted dose during the pre-patent phase should potentially reduce antimicrobial consumption in some specific cases where infections can be detected or predicted very early.

We assessed the effects of different infection-stage adjusted doses on clinical and microbiological outcomes by developing an original model of pneumonia in immunocompetent mice using *Pasteurella multocida*, which is one of the main pathogens responsible for respiratory diseases and pneumonia in cattle, swine and poultry. *Pasteurella* diseases are commonly treated at the group level with antibiotics of critical importance in human medicine, such as macrolides, fluoroquinolones and cephalosporins, and thus contribute to the overall consumption of these antibiotics in food-producing animals. This study mainly focused on beta-lactam antibiotics, which include “old” drugs such as amoxicillin and more recent drugs such as cefquinome, and which are known to have a net inoculum effect. This experimental model of infection is original in that it produces a mild-to-severe infection in immunocompetent animals, resulting in less than 100% prevalence of the clinical disease in challenged animals. These features were looked for in the model in order to mimic the prevalence of sick individuals following outbreaks of infection in groups of food-producing animals, which can range from 15 to 80% [14,16]. A system of non-invasive air-borne contamination was adopted in contrast to classical pneumonia models which consist of direct intra-tracheal or intranasal inoculation of the test pathogen under general anesthesia [22]. This more “natural” contamination route resulted in an initial highly reproducible bacterial load of about 10^4^ CFU/lung, associated with a slower development of the pulmonary infection, compared to intra-tracheal inoculation [7], with 60% of the mice expressing clinical signs 2 to 4 days after the challenge. We were thus able to compare at the group level the performances of an antibiotic treatment administered either to all mice during the pre-patent phase of the infection, characterized by the absence of visible/overt symptoms, or administered specifically to clinically affected mice (patent phase) with doses optimized for each situation. We hypothesized that the main biological determinant of the differences in antibiotic efficacy between the patent and pre-patent phases of infection would be the size of the bacterial load to be treated. Indeed, several works have shown that *in vitro* pathogen susceptibilities to many antibiotics are dependent on inoculum size [10,11], and that antibiotics are much more efficacious when low rather than high pathogen loads are targeted in rodent infection models [6,8,9,23,24]. Whereas all these previous studies involved direct inoculation of animals with initially low or high bacterial loads, we developed a model in which a low size inoculum was inoculated to all the animals and treatments were launched at different moments in the natural time-development of both bacterial load and clinical disease. In this context, we observed that during the pre-patent phase of infection, the bacterial inoculum in most mice with no clinical sign was below 5 log_10_ CFU, whereas during the patent phase of infection the bacterial inoculum in all mice was above 6 log_10_ CFU. We then decided to compare the outcomes associated with an antimicrobial treatment administered to all mice in the pre-patent phase, or only to those mice in the patent phase of infection, by using a dose specifically tailored to the pathogen load for each phase of the infection.

A pharmacokinetic study was carried out with each antibiotic to determine the fully curative dosage regimen for mice expressing clinical signs of infection (corresponding to the patent phase of the infection), and to calculate the doses required to attain a target value of T_>MIC_, the PK/PD index used for beta-lactams. Based on the commonly accepted value of T_>MIC_ = 50% for Gram negative bacteria [21,25,26], the calculated doses were 50 mg/kg/day for amoxicillin and 10 mg/kg/day for cefquinome. Three lower dosage regimens of each antibiotic, (2-fold, 10-fold and 20-fold lower) were also tested to identify an optimal dose for the pre-patent phase of infection. The ten-fold dose reduction was supported by our preliminary *in vitro* observations that amoxicillin was 8–9 times more potent against low (10^5^ CFU/mL) *versus* high (10^7^ CFU/mL) *P*.*multocida* inocula, and by *in vivo* observations using a rat model of *Klebsiella pneumonia* infection treated with cefquinome [12].

The data obtained for animals treated during the patent phase of infection firstly confirmed that T_>MIC_ was an efficient PK/PD index for predicting the efficacy of both amoxicillin and cefquinome. Indeed, a clinical cure rate of 100% was obtained with amoxicillin and cefquinome in animals expressing clinical signs of infection and with bacterial loads exceeding 10^6^ CFU/lung, using the PK/PD designed doses (50 and 10 mg/kg respectively) whereas the clinical cure rate was less than 85% with the half-doses corresponding to T_>MIC_ of 45 and 38%. For animals in the pre-patent phase of infection and harboring less than 10^5^ CFU/lung, a 100% clinical cure and a 90 to 100% microbiological cure rate were obtained with doses 10-fold lower than the PK/PD designed doses and corresponding to T_>MIC_ below 13% i.e. much lower than the breakpoint value of 50% of the dosage interval. Here again, the clinical cure rate was reduced to less than 80% by halving the doses and the microbiological cure rate to less than 60%. The fact that we got such high cure rates by administering 10-fold lower doses during the pre-patent phase of infection suggests that the PK/PD doses, optimized for moderate to severely infected individuals, are probably not optimal if individuals are treated at an earlier stage of infection. Assuming that the difference in efficacious dose reported for early and later interventions is mainly determined by the size of the pathogenic inoculum confronting the antibiotic, then our data and others [10,24] highlight the importance of factoring into the determination of PK/PD target values, which provide an efficient prediction of dosing regimen efficacy, the size of the bacterial load to be killed by the antimicrobial.

In veterinary medicine, the current collective treatments in food-producing animals actually correspond to a situation where different animals within the group are experiencing different phases of the infection time-course simultaneously. At the time of the decision to control an infectious disease, the groups of infected animals are usually composed of a minority of animals in the patent phase of infection (with overt clinical symptoms) and a majority of animals in the pre-patent phase of infection. In this study, we showed that by adjusting treatments to the infection phase, the individual dose required during the pre-patent phase could be reduced and at the same time avoid a negative impact on animal health by maintaining clinical efficacy. In addition, the impact on commensal microbiota might potentially be mitigated due to the reduction of their overall exposure to antimicrobial drugs. Indeed, we have previously demonstrated in a rat model of *K*. *pneumonia* infection that the doses of cefquinome needed to cure pre-patent infections prevented the amplification of ESBL-carrying Enterobacteria at the gut level whereas the doses needed to cure infection in the patent stage led, in all treated animals, to an amplification of ESBL-carrying Enterobacteria [12]. So, if the practicality of using doses adjusted to the pre-patent phase of infection is confirmed by further studies, we hypothesize that exposure of the intestinal tract to lower concentrations would help to maintain bacterial populations that are more susceptible to the administered drug and thereby prevent the emergence or selection of resistant populations.

Concerning antibiotic consumption, a 100% clinical cure was obtained either by treating 60 to 70% of the animals with a “standard” dose in the patent phase of infection or by treating 100% of the animals with a 10-fold lower dose in the pre-patent phase of infection. This means that the antibiotic consumption in our study was reduced 6-fold in the pre-patent phase treatments (metaphylaxis strategy) compared to the patent phase treatments. However, our “test” conditions were very favorable to these early treatments due to the higher percentage of clinically affected animals (60–70%) than under most field conditions [14,15], and to the fact that we launched an early treatment before any symptoms of infection were observed within the group. So, before implementing these results in the field, further studies in target species are needed to check that the classification of food-producing animals into patent or pre-patent phases is realistic and practicable, and that it does not lead to an increase of treatment failures in animals classified in the pre-patent phase.

However, even if a higher adjusted dose during the pre-patent phase than is theoretically possible would need to be used in field conditions, the gain in antimicrobial potency associated with the early treatments identified in this study would still largely improve current veterinary practices in terms of antibiotic consumption. The next required improvement in antibiotic stewardship will be to definitively stop the unnecessary use of treatments in the 20 to 70 percent of animals that escape an infection outbreak (either because they remain non-infected or are able to control the disease through natural immunity). With this ambitious perspective in mind, new treatment strategies relying on inexpensive methods of individual identification and early diagnosis, will therefore need to be proposed.

## Supporting information

S1 Table Amoxicillin and cefquinome plasma concentrations in mice. Observed plasma antibiotic concentrations *versus* time in mice (n = 3 at each time point) after a single subcutaneous administration of amoxicillin (50 mg/kg) or cefquinome (5 mg/kg). Cefquinome concentrations were below the LOQ for one mouse at 4 h, 2 mice at 6, 8 and 24 h and 3 mice at 12 h.
(XLSX)

S2 Table *Pasteurella multocida* counts in mouse lungs after an aerial challenge (raw data). Bacterial counts (log_10_ CFU/mL) and clinical status before the sacrifice are reported for each mouse at each sample time.
(XLSX)

S3 Table Clinical and microbiological cure rates with amoxicillin and cefquinome (raw data). Clinical and microbiological cure rates of mice after no treatment, early or late treatments with different doses of amoxicillin (2.5, 5, 25, or 50 mg/kg) or cefquinome (0.5, 1, 5, or 10 mg/kg). Rates were calculated for all the mice of the group in control and early treatments (as all the mice in a given group were subjected to the same protocol) whereas cure rates for the late treatments were only calculated from the 60 to 70% of mice treated with antibiotics. NA: not assessed.
(XLSX)
